# Significant increased isolation of Escherichia coli in Iranian women with endometriosis: a case control – study

**DOI:** 10.1186/s12905-024-03229-2

**Published:** 2024-07-03

**Authors:** Zohreh Tavana, Elham Askary, Mohammad Motamedi Far, Faranak Fatehpoor, Saeedeh Frooghinia, Alimohammad Keshtvarz Hesam Abadi, Kefayat Chamanara, Saeed Alborzi

**Affiliations:** 1 Obstetrics & Gynecology, Fellowship of infertility, Department of Obstetrics and Gynecology, School of Medicine, Infertility Research Center, Shiraz University Of Medical Sciences, Shiraz, Iran; 2 Obstetrics and Gynecology, Fellowship of Gynecologic Endoscopy, Department of Obstetrics and Gynecology, School of Medicine, Maternal-Fetal medicine Research Center, Shiraz University of Medical Sciences, Obstetrics and Gynecology Office, Shahid Faghihi Hospital, Zand Avenue, Shiraz, 7134844119 Iran; 3 Medical Microbiology, Department of Bacteriology and Virology, HIV/AIDS Research Center, Institute of Health, Shiraz University of Medical Sciences, Shiraz, Iran; 4 Obstetrics & Gynecologist, Department of Obstetrics and Gynecology, School of Medicine Shiraz University of Medical Sciences, Shiraz, Iran; 5 Fellowship of Gynecologic Endoscopy, Department of Obstetrics and Gynecology, Shiraz University of Medical Sciences, Shiraz, Iran; 6 Department of BioStatistics, Shiraz University of Medical Sciences, Shiraz, Iran; 7 Fellowship of Gynecologic Endoscopy, Department of Obstetrics and Gynecology, Shiraz University of Medical Sciences, Shiraz, Iran; 8 Saeed Alborzi, Department of Obstetrics and Gynecology, School of Medicine, Laparoscopy Research Center, Shiraz University of Medical Sciences, Shiraz, Iran

**Keywords:** Endometriosis, Endometritis, Culture, *Escherichia coli*

## Abstract

**Background:**

The role of bacterial contamination in the development and progression of endometriosis lesions is currently a hot topic for gynecologists. In this study, we decided to compare the endometrial cultures of women affected by endometriosis with those of non-endometriotic women, focusing on specific microbial pathogens.

**Material and method:**

In this cross-sectional case-control study, 30 women with endometriosis in stages 4 of the disease whose endometriosis was confirmed based on clinical, ultrasound, and histopathological findings, and 30 women without endometriosis who were candidates for surgery due to benign uterine diseases with regular menstrual cycle, underwent endometrial biopsy with Novak Kort in sterile conditions before starting their operation, and the results of their endometrial culture were analyzed and compared.

**Results:**

Results of the study indicate that there were no significant differences in terms of age, BMI, smoking, education level, place of residency, use of the intrauterine device, or vaginal douche, and age of menarche between the case and control groups. The only demographic difference observed was in parity, where the control group had a significantly higher parity than the case group (*P* = 0.001). Out of the 60 cultures, only 15 samples were positive in the endometriosis group, and E. coli was the most prevalent species, with 10 (33.3%) samples testing positive for it. Klebsiella spp. and Enterobacteria spp. were also detected in 3 (10.0%) and 2 (6.7%) samples, respectively. The comparison between the two groups showed that only E. coli had a significant association with the presence of endometriosis (*P* = 0.001). There was no significant relationship between the location of endometriosis in the pelvic cavity and culture results. It was observed that parity among the E. coli negative group was significantly higher compared to the E. coli positive group (*P* < 0.001).

**Conclusion:**

Based on The high occurrence of E. coli in women with endometriosis, along with its potential involvement in the progression and/or recurrence of this condition, the researchers propose that treating women with endometriosis and recurrent IVF failure, as well as those with endometriosis recurrence after surgical treatment, with suitable antibiotics and repeated culture until the culture becomes negative, could be beneficial.

## Introduction

Endometriosis is a chronic estrogen-dependent disease in which uterine glands and stroma are found outside the uterus. Endometriosis affects about 10% of women of reproductive age and 35–50% of women with infertility [1]. Its symptoms include dysmenorrhea, dyspareunia, dyschezia, dysuria, and infertility [2]. All of which lead to a decrease in the quality of life in affected women, so trying to find the possible pathophysiology in the development and progression of the endometriotic lesions is a hot topic for gynecologists today.

One of the proposed theories in endometriosis pathophysiology is the disorder of the immune system, genetic, and epigenetic processes [3]. In addition to the previous proposed theories, today, the role of bacterial contamination of endometrium in the pathogenesis of the disease has become more prominent. New recent studies have shown that the microbiota of the intestine, vagina, cervix, and endometrium of patients with endometriosis differs from that of healthy people. [1]. These microorganisms that lived in the vaginal cavity and adjacent organs such as the intestine or urinary tract, can spread to the upper genital tract through hematogenous or endogenous routes and cause infection or changes in host cell and organ behavior [4]. One of the most common bacteria that can migrate to the endometrial cavity and cause endometrial contamination is *E. coli* [5]. Due to the finding of endotoxin of *E. coli* species in the peritoneal cavity and menstrual fluid of endometriosis patients, infection with this bacterium has recently been proposed as an effective factor in the occurrence and growth of endometriosis lesions [6].

Exposure of peritoneal macrophages to (lipopolysaccharide, LPS) of E. coli as a gram negative bacterium, increases the synthesis of some macromolecules such hepatocyte growth factor (HGF), vascular endothelial cell growth factor (VEGF), interleukin (IL)-6, IL-8, and tumor necrosis factor-alpha (TNF-α). On the other hand, LPS by increasing the cell growth mediators can lead to significant proliferation of eutopic and ectopic epithelial and stromal endometrial cells [7]. Due to dynamic antibiotic resistance in this species, which has appeared over time, the use of standard antibiotic therapies in treating this bacterium which lives in the genitourinary & digestive system as a pathogenic flora, is unsuccessful [8].

Considering the role of bacterial infection in causing endometriosis, we decided to compare the cultures of endometriosis-affected women and women with other benign gynecological issues in terms of microbial factors in this study. So that we might open a new window in the process of treating the endometriotic patients with the aim of improving their quality of life, infertility, and preventing the recurrence of endometriotic lesions.

## Materials and methods

This cross-sectional, case-control study was carried out in the Women’s Department of Shiraz University of Medical Sciences in collaboration with the Bacteriology and Virology Department after approval in the ethics committee with the code IR.SUMS.MED.REC.1400.097. According to khan et al., study with considering the type I error (α = 0.05) and power (1-type II error = 90); 30 endometriotic patient, and 30 non-endometriotic women was selected as a case and control groups [6]. The patients were selected from women who were referred to the tertiary care center for laparoscopic surgery due to benign gynecological issues from June 2021 to December 2021. All participants filled out and signed the informed consent form before entering the study.

30 women with stage 3 and 4 endometriosis according to ASRM classification [9], confirmed through clinical, ultrasound, and histopathological findings, were compared with 30 non-endometriotic women with regular menstrual cycles. The latter group were candidates for surgery related to benign uterine conditions such as fibroid removal, endometrial polyps, or uterus removal due to issues like heavy menstrual bleeding, dysmenorrhea, or uterine enlargement. Inclusion criteria were women aged 18–45 years and regular menses (28 ± 7 days). Not using antibiotics, and any hormonal medications in the last 2 months, or using vaginal douches or vaginal medications or cervical treatments during the last 1 week, and not having sexual intercourse in the last 48 h, before surgery. Exclusion criteria were pregnancy, menopausal state, confirmed urogenital infection such as salpengitis, vaginitis, pelvic abscess, sexually transmitted disease, pelvic inflammatory disease, cervicitis, genital malignancy, inflammatory bowel disease, and body mass index > 30.

### Sample collection & culturing

Endometrial culture sample was obtained from all participants preoperatively and under anesthesia. The vagina and cervix were sterilized with povidine -iodine solution. The Novak curette was inserted through the cervical canal, negative pressure was applied by a 12 ml syringe attached to the instrument, and endometrial tissue was collected by aspiration and rotation and immediately transferred to buffer media. The culture was then sent to the Microbiology lab in cold box and subsequently evaluated. The primary culture was performed in MacConkey agar (MA), eosin methylene blue (EMB) agar, and tryptic soy agar (Himedia, India) under sterile condition and media were incubated at 37 °C for 24 h. For further investigation, the MA plates were maintained at 4 °C. Pure cultures were obtained. Identification of bacteria, cultural, morphological, and biochemical characteristics were studied. Then, the morphology and staining characteristics of bacteria were evaluated by Gram stain. Furthermore, biochemical tests, such as oxidase, sugar fermentation, catalase, methyl red (MR), Voges–Proskauer (VP), Simmon’s citrate, triple sugar iron, indole tests, urease and lysin decarboxylase, were carried out using standard methods [10]. Cultures positive for *Enterobacter spp.*, *Klebsiella spp.*, and *E. coli* were checked and recorded.

### Statistical analysis

The results of study groups were analyzed by SPSS (Version 22.0; SPSS Inc., Chicago, IL, USA) software. The Kolmogorov-Smirnov test was used to evaluate the normality of continuous data. Continuous variables were displayed as mean ± standard deviation and median (interquartile range), while categorical variables were presented as number (percentage). To compare continuous and categorical variables among study groups, Mann-Whitney and Fisher’s exact tests were used, respectively. *P* < 0.05 was considered to indicate a statistically significant difference.

## Results

The average age of the participant in our study was 37.45 year (SD: 5.47, Range: 24–45). The average BMI was 27.17 (SD: 2.29; range: 22–30), and the average of menarche age was 12.07 year (SD: 1.12; range: 10–14). As shown in Table 1, there was no difference in terms of age, BMI, smoking, education level, place of residency, use of intrauterine device, or vaginal douche and age of menarche between the case and control groups. The only demographic difference between the two groups was in parity, and the parity of the control group was significantly higher than the case. (*P* = 0.001)

**Table 1:** Comparison of demographic and clinical characteristics of endometriosis patients (cases) and the control group

Variable		Total; *N* = 60	Group		*P*-value
			Case; *N* = 30	Control; *N* = 30	
Age (years)-					0.101
Mean ± Standard Deviation,		37.45 ± 5.47,	38.87 ± 4.17,	36.03 ± 6.28,	
Median (Interquartile Range)		38.0 (8.0)	39.50 (6.50)	37.0 (9.25)	
BMI (kg/m^2^)-					0.606
Mean ± Standard Deviation,		27.17 ± 2.29,	27.00 ± 2.36,	27.33 ± 2.23,	
Median (Interquartile Range)		28.0 (3.0)	27.50 (4.0)	28.0 (3.0)	
Parity-					0.001
Mean ± Standard Deviation,		2.03 ± 1.50,	1.37 ± 1.25,	2.70 ± 1.50,	
Median (Interquartile Range)		2.0 (2.0)	1.0 (2.0)	2.50 (1.50)	
Menarche (years)-					0.836
Mean ± Standard Deviation,		12.07 ± 1.12,	12.10 ± 1.16,	12.03 ± 1.10,	
Median (Interquartile Range)		12.0 (2.0)	12.0 (2.0)	12.0 (2.0)	
Education, N (%)	Illiterate	2 (3.3)	1 (3.3)	1 (3.3)	0.723
	Under-diploma	9 (15.0)	4 (13.3)	5 (16.7)	
	Diploma	21 (35.0)	13 (43.3)	8 (26.7)	
	Bachelor	20 (33.3)	8 (26.7)	12 (40.0)	
	Masters	8 (13.3)	4 (13.3)	4 (13.3)	
Residence, N (%)	Urban	24 (40.7)	12 (40.0)	12 (41.4)	0.854
	Sub-urban	14 (23.7)	10 (33.3)	11 (37.9)	
	Rural	21 (35.6)	8 (26.7)	6 (20.7)	
Smoking, N (%)	Positive	8 (13.3)	4 (13.3)	4 (13.3)	> 0.999
	Negative	52 (86.7)	26 (86.7)	26 (86.7)	
Vaginal Shower, N (%)	Positive	7 (11.7)	3 (10.0)	4 (13.3)	0.688
	Negative	53 (88.3)	27 (90.0)	26 (86.7)	
IUD, N (%)	Yes	19 (31.7)	7 (23.3)	12 (40.0)	0.165
	No	41 (68.3)	23 (76.7)	18 (60.0)	

BMI: Body mass index; IUD: Intrauterine device

All endometriosis patients were in the stage 4 of endometriosis. The average endometriosis score in the endometriosis group was 123.47 ± 48.08.

According to Table 2, out of all 60 cultures collected from two groups of patients, only 15 samples were positive in the endometriosis group, which was as follows: 10 (33.3%) sample with *E. coli*, 3 (10.0%) with *Klebsiella spp.*, and two (6.7%) with *Enterobacteria spp*. Based on the comparison between two groups, only E. coli species had significant association with the presence of endometriosis (*P* = 0.001).

**Table 2:** Frequency of cultures positive in the studied groups

	Culture positive	Total Number = 60	Endometriosis group Number = 30	Non-Endometriosis group Number = 30	*P*-value between groups
**Bacterial species**	***E. coli***	10 (16.7)	10 (33.3)	0	**< 0.0001**
	***Klebsiella***	3 (5.0)	3 (10.0)	0	0.08
	***Enterobacter***	2 (3.3)	2 (6.7)	0	0.15

Among the cultures collected from two groups, 15 samples were positive, and all of them belonged to the endometriosis group. Based on the microbiological assays, 10 (33.3%) were *E. coli*, 3 (10.0%) were *Klebsiella spp.*, and two (6.7%) were *Enterobacteria spp*. Only endometrial contamination with *E. coli* was significantly associated with endometriosis (*P* = 0.001).

The cultures of the endometriosis group were evaluated regarding the demographic factors in our study (Tables 3 and 4). As demonstrated, only parity among the *E. coli* negative group was significantly higher compared to *the E. coli* positive group (*P* < 0.001).

**Table 3:** Evaluation of microbiological culture results based on continuous demographic and clinical features of endometriosis patients

Variable	Endometriosis; *N* = 30	Culture; [positive vs. negative]					
		E.Coli; *N* = 10	*P*-value	Klebsiella; *N* = 3	*P*-value	Enterobacter; *N* = 2	*P*-value
Age (years)- Mean ± Standard Deviation, Median (Interquartile Range)	38.87 ± 4.17, 39.50 (6.50)	40.10 ± 3.98 vs. 36.92 ± 5.61, 41.50 (8.25) vs. 38.50 (5.75)	0.097	40.33 ± 1.15 vs. 37.30 ± 5.58, 41.0 () vs. 38.0 (8.0)	0.424	39.00 ± 2.83 vs. 37.40 ± 5.55, 39.0 () vs. 39.50 (7.50)	0.824
BMI (kg/m^2^)- Mean ± Standard Deviation, Median (Interquartile Range)	27.00 ± 2.36, 27.50 (4.0)	26.70 ± 2.83 vs. 27.15 ± 2.16, 27. 0 (5.25) vs. 28.0 (3.75)	0.756	26.00 ± 2.00 vs. 27.11 ± 2.41, 26.0 () vs. 28.0 (4.0)	0.364	26.50 ± 0.71 vs. 27.04 ± 2.44, 26.0 () vs. 28.0 (4.0)	0.615
Parity- Mean ± Standard Deviation, Median (Interquartile Range)	1.37 ± 1.25, 1.0 (2.0)	0.60 ± 0.70 vs. 2.32 ± 1.45, 0.50 (1.0) vs. 2.0 (2.0)	**< 0.001**	2.00 ± 1.73 vs. 2.04 ± 1.50, 3.0 (3.0) vs. 1.0 (2.0)	0.876	1.00 ± 1.41 vs. 2.07 ± 1.50, 1.0 () vs. 1.0 (2.0)	0.366
Menarche- Mean ± Standard Deviation, Median (Interquartile Range)	12.10 ± 1.16, 12.0 (2.0)	12.00 ± 1.05 vs. 12.15 ± 1.23, 12.0 (2.0) vs. 12.0 (2.0)	0.666	12.00 ± 1.19 vs. 12.11 ± 1.00, 12.0 () vs. 12.0 (2.0)	0.887	12.00 ± 1.41 vs. 12.11 ± 1.17, 12.0 () vs. 12.0 (2.0)	0.898
Score- Mean ± Standard Deviation, Median (Interquartile Range)	123.47 ± 48.08, 131.0 (74.0)	121 0.20 ± 64.71 vs. 124.60 ± 39.25, 141.0 (127.0) vs. 129.0 (63.0)	0.930	166.00 ± 12.49 vs. 118.74 ± 48.32, 170.0 () vs. 128.0 (82.0)	0.057	116.00 ± 65.05 vs. 124.00 ± 48.19, 116.0 () vs. 131.0 (65.0)	0.901

BMI: Body mass index

**Table 4:** Evaluation of microbiological culture results based on categorical demographic and clinical features of endometriosis patients

Variable		Endometriosis; *N* = 30	Culture positive					
			E.Coli; *N* = 10	P-value	Klebsiella; *N* = 3	P-value	Enterobacter; *N* = 2	P-value
Education	Illiterate	1 (3.3)	1 (10.0)	0.376	0 (0)	0.245	0 (0)	> 0.999
	Under-diploma	4 (13.3)	1 (10.0)		1 (33.3)		0 (0)	
	Diploma	13 (43.3)	5 (50.0)		0 (0)		1 (50.0)	
	Bachelor	8 (26.7)	1 (10.0)		2 (66.7)		1 (50.0)	
	Masters	4 (13.3)	2 (20.0)		0 (0)		0 (0)	
Residence	Urban	12 (40.0)	5 (50.0)	0.386	0 (0)	0.167	1 (50.0)	0.724
	Sub-urban	10 (33.3)	1 (10.0)		2 (66.7)		1 (50.0)	
	Rural	8 (26.7)	4 (40.0)		1 (33.3)		0 (0)	
Smoking	Positive	4 (13.3)	2 (20.0)	0.584	0 (0)	> 0.999	0 (0)	> 0.999
	Negative	26 (86.7)	8 (80.0)		3 (100)		2 (100)	
Vaginal Shower	Positive	3 (10.0)	1 (10.0)	> 0.999	0 (0)	> 0.999	0 (0)	> 0.999
	Negative	27 (90.0)	9 (90.0)		3 (100)		2 (100)	
IUD	Yes	7 (23.3)	1 (10.0)	0.148	1 (33.3)	> 0.999	0 (0.0)	> 0.999
	No	23 (76.7)	9 (90.0)		2 (66.7)		2 (100)	

N (%);IUD: Intrauterine device

Also, according to Table 5, endometriosis patients were examined and compared in terms of the location of pelvic involvement with endometriosis lesions, and no significant relationship was found between the location of endometriosis in the pelvic cavity and culture results.

**Table 5:** Evaluation of microbiological culture results based on endometrioma, hydrosalpinx, and rectal DIEs

Pelvis involvement		Number of case	E.coli		*P* value	Klebsiella; *N* = 3		*P*	Enterobacter; *n* = 2		*P* value
			Positive *N* = 10	Negative *N* = 20		Positive *N* = 3	Negative *N* = 27		Positive *N* = 2	Negative *N* = 28	
Endometriomas	Negative	3 (10.0)	1 (10.0)	2 (10)	> 0.999	0 (0)	3 (11.1)	value > 0.999	1 (50.0)	2 (7.1)	0.193
	Positive	27 (90.0)	9 (90.0)	18 (90)		3 (100)	24 (88.9)		1 (50.0)	26 (92.9)	
Hydrosalpinx	Negative	13 (43.3)	6 (60.0)	7 (35)	0.255	1 (33.3)	12 (44.4)	> 0.999	0 (0.0)	13 (46.4)	0.492
	Positive	17 (56.7)	4 (40.0)	13 (65)		2 (66.7)	15 (55.6)		2 (100.0)	15 (53.6)	
Rectal DIE	Negative	18 (60.0)	4 (40.0)	14 (70)	0.139	2 (66.7)	16 (59.3)	> 0.999	0 (0)	18 (64.3)	0.152
	Positive	12 (40.0)	6 (60.0)	6 (11)		1 (33.3)	11 (40.7)		2 (100)	10 (35.7)	

N (%); DIE: deep infiltrating endometriosis

## Discussion

In the present study, based on the comparison made between two groups of endometriosis and women with other benign gynecological issues, positive *E. coli* culture in endometrial samples was significantly higher in women with endometriosis compared to the control group. (*P* = 0.001), and the parity among patients with endometriosis and positive E.coli cultures was significantly lower than those with negative cultures in the control, and case groups (*P* < 0.001).

One important aspect of this study is the use of a culture-based approach to assess bacterial contamination in the uterine cavity through endometrial tissue biopsy samples. Previous studies have utilized various methods to investigate bacterial contamination in women with endometriosis, such as measuring bacterial endotoxin levels in menstrual blood and peritoneal fluid [6, 7], using endometrial swabs [12], and identifying microbiomes through DNA sequencing of the 16 S rRNA marker gene [13], but all these methods have certain limitations such lack of sample trail for repeat testing and antibiotic susceptibility testing, low taxonomical resolution, and costly laboratory setup and maintenance are other limitations of these technologies [14–16] While nowaday the NGS (next-generation sequencing) method is considered more sensitive than culture in identifying microorganisms, particularly in non-cultivable and fastidious bacteria [14], NGS is less specific and raises concerns about the potential for false amplification, leading to positive results [17]. Furthermore, its higher cost is a significant consideration, especially for middle- to low-income countries [16], making it currently not advisable for routine use. Therefore, bacteriological culture from an endometrial biopsy provides the most accurate results, surpassing even swab sampling in terms of sensitivity and positive predictive value [18]. Additionally, it is affordable and accessible.

In 2018, Khan et al. examined bacterial endotoxin levels in menstrual blood and peritoneal fluid and their potential impact on endometriosis development. Their study revealed that E. coli LPS contributes to regulating pelvic pro-inflammatory responses and the progression of endometriosis through the LPS/TLR4 cascade. Patients’ menstrual blood cultures showed a significant presence of E. coli. This study introduced the “bacterial contamination hypothesis” for endometriosis patients, proposing a potentially beneficial new treatment alongside conventional therapies for the disease [7] Like preceding researches, in our study, the endometrium of women with advanced stages of endometriosis was highly contaminated by E. coli.

In a 2019 systematic review by Koninckx P. et al., women with endometriosis had a notably higher risk of urinary tract infections, chronic endometrial inflammation, severe pelvic inflammation, and infection at the surgical site following hysterectomy. E. coli was one of the most common organisms causing these infections [19].

Wei et al. conducted a study that found a significant statistical difference in the diversity of bacteria in the cervical mucus of endometriosis patients. The diversity gradually increases towards the upper parts of the reproductive system [20]. According to the study, Lactobacillus decreased significantly in the upper reproductive tract of endometriotic patients, with type IV flora becoming dominant. Community diversity increased as a result of Pseudomonas, Acinetobacter, and Vagococcus in these patients. The researchers concluded that the distribution of genital tract flora differs between the upper and lower reproductive tract in endometriotic patients compared to non-endometriotic subjects [20].

Despite all these findings, proving the relationship between infection and endometriosis, and determining which is the cause and which is the result, is challenging due to the presence of various microbial agents in the reproductive system [21].

There are two types of E. coli: intestinal and non-intestinal. Generally, the intestinal *E. coli* bacteria are phylogenetically distinct from non-intestinal ones [22]. The intestinal type is responsible for diseases such as UTI, neonatal meningitis, Wound infection [23, 24], and female genital tract infection [25], especially in women with estrogen withdrawal, there is an increase in vaginal pH, and absence of vaginal lactobacilli [23].

Cook’s study found that E. coli isolated from the female genital tract and neonatal sepsis have unique characteristics that increase their virulence. Research has shown that uropathogenic Escherichia coli (UPEC) is the most significant cause of UTIs and can invade the vaginal epithelium as an extra-intestinal pathogen [26].

UPEC is grow in urine and other extra-intestinal areas with their particular virulence factors. UPEC virulence factors placed on the plasmid or at specific chromosome points are called “pathogenicity islands. Resistance to various classes of common antibiotics among UPEC strains has become a significant concern in treating UTIs caused with UPEC species [25].

Several studies have supported the hypothesis that changes in the endometrial microbiota of infertile women may affect their reproductive potential.

Kim et al. found that vaginal colonization with Gram-negative bacteria, especially E. coli, was significantly higher in pregnancies following infertility treatment [27]. Xu et al. demonstrated that tubal obstruction, prolonged menstrual cycle, and vaginal pH > 4.5 are all linked to increased colonization of E. coli in the vagina [28]. Safarpour and their colleagues assessed UPEC strain properties isolated from high vaginal swab samples in fertile and infertile women. They found that resistant and virulent UPEC strains have a higher prevalence in the upper vaginal areas of infertile women with a history of urinary tract infection, indicating the important role of these microorganisms in causing female infertility [29]. In this regard, Zhang et al. utilized 16 S rRNA gene sequencing to demonstrate that the endometrial microbiome of infertile women experiencing repeated implantation failure (RIF) or recurrent pregnancy loss (RPL) consists of diverse bacteria, such as E. coli [30].

Based on the aforementioned studies, in our study, we found that endometriosis patients with negative E. coli cultures had a significantly higher parity compared to those with positive cultures. This could contribute to the higher infertility rate among endometriosis patients with a positive E. coli culture. However, we did not find a significant relationship between hydrosalpinx and bacterial culture in our study.

The study by Farsimadan et al. suggests that even spermatozoa can carry various bacteria, potentially leading to contamination of the oocyte. This contamination can disrupt fertilization, result in low embryo quality, and contribute to ART failure [11]. So, identifying endometrial dysbiosis as a potential cause of infertility could be a step toward improving treatment for infertility patients [28]. It is also worth considering E.coli as a potential asymptomatic STD infection that can impact male and female fertility, and as an epigenetic factor that may contribute to the progression and/or recurrence of endometriosis lesions.

Limitations of this study are the small sample size, failure to examine only infertile patients in two groups, and absence of antibiogram data; these aspects could be considered in future research.

## Conclusion

Given the high occurrence UPEC E. coli in women with low parity and endometriosis, as confirmed in our study, along with the potential involvement of E. coli in the progression and/or recurrence of these conditions, we propose that treating women with endometriosis, especially in those attempting to conceive, with suitable antibiotics and repeated culture, followed by subsequent antibiotic susceptibility testing until their culture becomes negative, could be advantageous.

## Data Availability

The datasets used and/or analyzed during the current study are available from the corresponding author on reasonable request.
